# Coupling and Coordinated Development of Higher Vocational Education and Economic Development in the Yangtze River Economic Belt from 2008 to 2020

**DOI:** 10.1155/2022/2643635

**Published:** 2022-09-09

**Authors:** Xiao Ren, Yingling Zhou

**Affiliations:** ^1^Chongqing Vocational College of Culture and Arts, Chongqing 401320, China; ^2^Research Center for Economy of Upper Reaches of Yangtze River, Chongqing Technology and Business University, Chongqing 400067, China; ^3^Green Development Research Institute, Chongqing Finance and Economics College, Chongqing 401320, China

## Abstract

According to the panel data from 2008 to 2020, empirical research is conducted on the coupling and coordination relationship between the higher vocational education and the regional economic development in the Yangtze River economic belt by constructing a comprehensive evaluation indicator system and using a coupling and coordination degree model. The research results show that, the development levels of the higher vocational education system and the regional economic development system in the Yangtze River economic belt have been on the rise over the past 13 years. There existed a sound coupling interaction between the two systems. However, their coupling and coordination degree were not high. By the end of 2020, 73% of the provinces (cities) were in transition and imbalance, and the coupling coordination degree showed a gradient phenomenon of “high in the East and low in the west.” To improve the coupling and coordination level between the two systems, the Yangtze River Economic Belt shall deepen the supply-side reform of the higher vocational talent training in accordance with the needs of industrial development, optimize the layout of higher vocational specialties to boost the adaptability of education to economy, and strengthen regional overall coordination to accelerate the cluster development of regional higher vocational education.

## 1. Introduction

As an important part of national education system and human resources development, the higher vocational education is the education type, which is most closely related to the economic and social development, as well as an important support to promote the effective connection between education chain, talent chain, industrial chain, and innovation chain. On the one hand, the higher vocations are closely linked to the restructuring, transformation, and upgrading of industrial structure, which can provide indispensable manpower and intellectual support for economic development. On the other hand, the regional economic development plays the role of baton for optimizing specialty setup and innovating talent training mode in the higher vocational education, which determines scale, quality, and development speed of regional higher vocational education to a certain extent. Higher vocational education and regional economic development are a community of common destiny that embed and promote each other [1]. In this context, how to give full play to the advantages of the higher vocational education to promote the high-quality development of the regional economy is one of the issues that need to be considered urgently. In 2016, the outline of the Yangtze River economic belt development plan was officially issued, and China's promotion of the construction of the Yangtze River economic belt entered an accelerated period. As the region with the most vitality and potential for China's economic and social development, the significance of promoting the development of the Yangtze River economic belt is self-evident. Covering 11 provinces and cities, such as Shanghai, Jiangsu, and Zhejiang, the Yangtze River economic belt spans the three major regions of the east, middle, and west of China, accounting for about 21% of the country's area, and its population and GDP exceed 40% of the country. It is one of the regions with the strongest comprehensive strength and the largest strategic support in China [2]. Therefore, this research explores the development level of and the coupling and coordination relationship between the higher vocational education system and the regional economical development system of the 11 provinces (cities) in the Yangtze River economic belt by constructing the coupling and coordination model, with view to providing theoretical support and empirical evidence for promoting the high-level coordinated development of higher vocational education and social economy in the Yangtze River economic belt.

## 2. Literature Review

Education and economy have always been two inseparable words, closely related and complementary, which have drawn long-term attention from academic circles at home and abroad. As early as the 1960s, Theodore W. Schultz blazed a new path for the research on the relationship between education and economic growth based on his human capital theory. According to such theory, compared with the contribution of physical capital to the economy, the systematic improvement of human resources is significantly effective, while education is the main way of human capital accumulation [3]. Later, scholars represented by Paul Romer and Robert Lucas further explained that knowledge and specialized human capital are the main sources and driving forces of economic growth, stressing that education boosts economic growth while accelerating technological progress [4]. Herbertsson found according to his empirical research on the reasons for the economic growth of the Nordic countries that the most significant factor contributing to the economic growth is the human resources with systematic and formal education [5]. Pereira proved the difference in the contributions of different educational inputs to economic growth by measuring the impact of human resources at various levels of education on the economic growth level [6]. It can be seen that related overseas research mostly focused on one-way contribution and impact of education to economic development, and most of the conclusions show that education positively accelerates economic development.

Compared with overseas research, domestic research pays more attention to the interaction between education and economy. The existing pieces of research mainly focus on theoretical discussion and empirical analysis. The theoretical discussions mainly focus on the inner link and coordination path between education and economic development. For example, He et. al. analyzed the dialectical relationship of the two-way drive between education and economy using the logical structure diagram and proposed policy recommendations based on practices [7]. Wang analyzed the typical experience of Germany, the United Kingdom, Australia, and other developed countries in ensuring the coordinated development of vocational education and economy and proposed some initiatives for the coordinated development of the two from the perspectives of institutional level, organization, and specific strategy [8]. Xia and Shi analyzed mutual acceleration between the higher vocational education and the regional economic development from the perspective of the integration of production and education and proposed 5 paths for coordinated development, for example, broadening the idea of running schools and implementing coordination management [9]. Taking 31 provinces and cities in China as cases, Wang and others applied configuration thinking and fsQCA method to explore the complex causal mechanism that affects the high-quality development of regional economy by integrating the internal and external conditions of higher education [10].In terms of the empirical analysis, many pieces of research on the coordinated development relationship between education and economy are conducted using the econometric model, statistical analysis, and other methods. For example, Zhou and Zhu analyzed the relationship between the higher vocational education and the economic development level in Chongqing based on the time series data using the Granger causality test method and proposed a path to construct a coordinated development mechanism [11]. Fu and Zhao obtained the spatial-temporal differentiation of higher education and economic development in 31 provinces (cities) of China under the background of “Double First-class Initiative” using the coordination degree model, ESDA method, and gray correlation method and proposed the policy recommendation of strengthening the coordinated development of higher education and economy [12]. Hao conducted an empirical analysis on the spatial-temporal stability of the coordination between higher education and regional economy in China from 2009 to 2018 using the spatial statistics method and came to the conclusion that higher education and regional economy are interdependent and complementary [13]. Xie and others analyzed the dynamic relationship between higher vocational education investment and industrial structure upgrading by establishing a PVAR model and using the panel data of 31 provinces in China from 2009 to 2018. They concluded that higher vocational education investment has a positive effect on industrial structure upgrading in the short term, and the promotion effect of industrial structure upgrading on higher vocational education investment shows a phased characteristic of “positive and negative alternation” in the short term [14].

From the perspective of research, scholars focus more on the relationship between the undergraduate education or the entire higher education and the economic system but less on the higher vocational education. From the perspective of research samples, scholars focus more on the national or single provincial level but less on coupling and coordination between higher vocational education and the economy system at the meso level, especially in the Yangtze River economic belt. From the perspective of research methods, scholars focus more on the causality test method, spatial statistics method, and other econometrics or statistics method. Such methods will inevitably be interfered by subjective factors. The coupling and coordination model based on the objective assignment of the entropy method can avoid subjective randomness. Therefore, based on the panel data of the 11 provinces (cities) in the Yangtze River economic belt, including Shanghai, Jiangsu, Zhejiang, Anhui, Jiangxi, Hubei, Hunan, Chongqing, Sichuan, Guizhou, and Yunnan from 2008 to 2020, this research measured comprehensive development level and coupling and coordination degree of the higher vocational education and economic development system, which has important practical significance for analyzing the current situation of the coordinated development of the two and accelerating the high-level coordinated development of the two.

## 3. Construction of Indicator System and Data Sources

### 3.1. Construction of Evaluation Indicator System

On the basis of sorting out relevant research literature, according to connotation and characteristics of the higher vocational education and the regional economic development, in combination with scientificity, systematicness, hierarchy, feasibility, and other principles for constructing the indicator system, a comprehensive evaluation indicator system for the higher vocational education system and the regional economic development system is constructed.

#### 3.1.1. Construction of the Higher Vocational Education Evaluation Indicator System

As an education type different from general education, the vocational education has both the higher education attributes and distinct occupation (employment) orientation. Based on the perspective of “input-process-output” educational performance evaluation [15], the higher vocational education system selected the education scale, education quality, and education achievement as criterion-level consideration dimensions. The education scale is a description of the basic situation of education, which reflects the degree of educational development, including three evaluation indicators, namely the number of higher vocational colleges, enrolments, and the number of students at colleges [16]. The education quality depends on human and financial supports. In vocational colleges, education quality is mainly reflected in the investment and allocation of education funds and teachers [17]. In this research, education quality is mainly reflected in the three indicators, namely national fiscal education fund, educational income, and teacher-student ratio. The education achievement is a result evaluation indicator that reflects the efficiency of education investment. Combined with college-running orientation and objective of the higher vocational colleges, education achievement in this research is reflected by the following three indicators: the number of granted patents of higher vocational colleges, number of awards in National Competition for Skills of Vocational Education (higher vocational college group), number of national model (backbone) and “high-level higher vocational college with Chinese characteristics and specialty construction plan” higher vocational colleges.

#### 3.1.2. Construction of Regional Economic Development Evaluation Indicator System

According to the characteristics of regional economic development, the regional economic development system selected economic scale, economic structure, economic benefit, and innovative development as criterion-level consideration dimensions. The economic scale is a description of integrated capacity and development situation of the regional economy, which is reflected in this research by the following two indicators: per capita gross regional product and employees in all sectors of society. The economic structure reflects the distribution of social productivity and production factors, which is reflected in this research by the following two indicators: the proportion of secondary industry in GDP and the proportion of tertiary industry in GDP. The economic benefit reflects the relationship between social and economic input and output, which is reflected in this research by the three indicators: per capita income gap between urban and rural areas, GDP growth rate, and Engel coefficient. The innovative development reflects the potential and development trend of local economic growth and depends on the investment in labor forces and funds for scientific and technological innovation [18], which is reflected in this research by the following three indicators: the internal expenditure of research & development (R&D) fund, the full-time equivalent of research & development (R&D) personnel, and the number of granted patents.

### 3.2. Description of Data Sources

The data sources of the higher vocational education system in this research are Educational Statistics Yearbook of China 2008–2020, China Educational Finance Statistical Yearbook 2009–2021, local statistical yearbooks of the provinces (cities) in the Yangtze River economic belt, and the official website of the Ministry of Education. The number of national model (backbone) and “high-level higher vocational college with Chinese characteristics and specialty construction plan” higher vocational colleges is composed of national model higher vocational college construction units in 2006, national backbone higher vocational college construction units in 2010, and high-level higher vocational colleges with Chinese characteristics and specialty construction plan units in 2019. The data sources of the regional economic development system are China Statistical Yearbook 2009–2021, China Statistical Yearbook on Science and Technology 2009–2021, and local statistical yearbooks. In this research, the entropy method is used to determine the weights of indicator data. The indicator system and the weight calculation results are shown in Table 1.

## 4. Research Method Design

### 4.1. Entropy Method

In thermodynamics, entropy is a measuring unit of irregularity and randomness in a closed system. According to its characteristics, the importance of an indicator can be determined based on the dispersion degree of the indicator reflected by the information entropy. The higher the dispersion degree, the greater the impact of the indicator on the comprehensive evaluation, and vice versa. Compared with the subjective weighting evaluation method, the entropy method uses the principle of information entropy to objectively determine the weight, which can better avoid the subjective randomness of weighting in the calculation program and conduct the object evaluation more accurately and objectively, which is suitable for the comprehensive evaluation of multiple indicators in this study [19]. Therefore, the entropy method is used for the weight calculation in this research. The specific steps are as follows:

(1) Standardize the indicators. To eliminate dimension and unit difference between the evaluation indicators of the higher vocational education system and the regional economic development system, the indicator data shall be standardized. The function model is as follows:
  (1) $$\begin{array}{c} {X^{\prime }}_{i\theta j}=\left\{\begin{array}{cc} \frac{x_{i\theta j}-x_{j,\min}}{x_{j,\max}-x_{j,\min}} & x_{i\theta j}is\;positive\;in\;cator,\\ \frac{x_{j,\max}-x_{i\theta j}}{x_{j,\max}-x_{j,\min}} & x_{i\theta j}is\;positive\;in\;di\;cator\text{.} \end{array}\right. \end{array}$$
Assume that that there are *m*years, *r*provinces (cities), and *n*indicators. *x*_*iθj*_is *jth* indicator value of province (city)*θ* in *ith* year in the evaluation indicator. *x*_*j*,max_ and*x*_*j*,min_ are, respectively, maximum and minimum values of *jth* indicator. *r*=1,2,3 ⋯ *m*(number of evaluation years), *θ*=1,2,3 · ··*r*(number of provinces/cities), and *j*=1, 2, 3 · ··*n*(number of evaluation indicators). *X*_*iθj* _′ is the value after standardization, and the value range is limited to [0,1]. To avoid the special situation of “0” evaluation indicator value after standardization to ensure the scientificity and effectiveness of evaluation indicators, all the evaluation indicators are subjected to translation processing after standardization, and the function model is as follows:
  (2) $$\begin{array}{c} \begin{array}{c} X_{i\theta j}={X^{\prime }}_{i\theta j}+0.001 \end{array}\text{.} \end{array}$$
(2) Calculate the weight of each indicator. On this basis, the entropy method is used to calculate the weight value of each indicator in the higher vocational education system and the regional economic development system. The function model is as follows:
  (3) $$\begin{array}{c} P_{i\theta j}=\frac{X_{i\theta j}}{\sum_{i=1}^{m}\sum_{\theta =1}^{r}X_{i\theta j}}\text{,} \end{array}$$
  (4) $$\begin{array}{c} Ej=-k\overset{m}{\underset{i=1}{\sum }}\overset{r}{\underset{\theta =1}{\sum }}P_{i\theta j}\ln P_{i\theta j}\text{,} \end{array}$$
  (5) $$\begin{array}{c} G_{j}=1-E_{j}\text{,} \end{array}$$
  (6) $$\begin{array}{c} M_{j}=\frac{G_{j}}{\sum_{j=1}^{n}G_{j}}\text{.} \end{array}$$

Equation (3) is used to determine the proportion of each evaluation indicator value, where*P*_*iθj*_refers to the proportion of *j*^*th*^ indicator value of the province (city)*θ* in*i*^*th*^ year to this type of indicator. Equation (4) is used to calculate the entropy of each indicator, *E*_*j*_ is the entropy of *j*^*th*^ indicator, where constant *k*=1/ln(*mr*). Equation (5) is used to calculate the information utility value of *j*^*th*^ indicator, and equation (6) is used to calculate the weight of each evaluation indicator.

### 4.2. Coupling Degree Model

The coupling degree refers to the degree of information or parameter dependence between modules. The coupling degree calculation is a way to measure the degree of correlation between systems. When there exists a relationship of mutual integration and promotion between the two systems, the coupling between the two systems is good. When there exists mutual constraint and restriction between the two systems, the coupling between the two systems is poor [20]. The coupling degree function model of the higher vocational education system and the regional economic development system, based on the relevant assumptions of the coupling theory, is as follows:

(7) $$\begin{array}{c} T_{i}=\overset{n}{\underset{j=1}{\sum }}\delta_{ij}X_{ij},\overset{n}{\underset{j=1}{\sum }}\delta_{ij}=1\;i=1,2\text{,} \end{array}$$

(8) $$\begin{array}{c} C=2{\left[\frac{T_{1}\times T_{2}}{{\left(T_{1+}T_{2}\right)}^{2}}\right]}^{1/2}\text{.} \end{array}$$

In equations (7) and (8), let *T*_1_ represent the comprehensive evaluation indicator of the higher vocational education system and *T*_2_ represent the comprehensive evaluation indicator of the regional economic development system. *X*_*ij*_ is the assignment of each indicator in the two systems after standardization, *δ*_*ij*_ is the weight coefficient of each indicator in the two systems, and the coupling degree value between the two systems is*C* ∈ [0, 1].

Referring to the existing pieces of research, the coupling status can be divided into several stages according to the coupling degree value. When *C* value is 1, the coupling level is the highest, indicating that the coupling between the two systems is good and develops in an orderly manner. When 0.8 < *C* < 1, the two systems are in a high-level coupling status. When 0.5 < *C* ≤ 0.8, the two systems are in the running-in stage. When 0.3 < *C* ≤ 0.5, the two systems are in a rival stage. When 0 <*C* ≤ 0.3, the two systems are in a low-level coupling stage. When*C*value is 0, the coupling level is the lowest, indicating that the two systems are in an unrelated and disordered status [21].

### 4.3. Coupling and Coordination Degree Model

The coupling degree only measures the degree of interaction between the systems, however, it is hard to reflect the coordination status and development level of the higher vocational education system and the regional economic development system. The coupling and coordination degree can represent the degree of coordinated development between the systems and the level of development [22]. Therefore, a coupling and coordination degree model is constructed to further determine the coordinated development level of the higher vocational education system and the regional economic development system on the basis of the coupling degree. The function model is as follows:

(9) $$\begin{array}{c} F=\sqrt{C\times \left(\lambda T_{1}+\mu T_{2}\right)}\text{.} \end{array}$$

In equation (8), *F* is the coupling and coordination degree, *C* is the coupling degree, *λT*_1_+*λT*_2_ is the coordinated development parameter, *T*_1_ and*T*_2_ are, respectively, the comprehensive evaluation indicators of the higher vocational education system and the comprehensive evaluation indicator of the regional economic development system, *λ* and *μ* are undetermined coefficients, respectively, representing the importance of higher vocational education and regional economy in coordinated development, where *λ*+*μ*=1. Considering the mutual promotion and equal importance of higher vocational education and the regional economic development, this research assigns *λ* and *μ* as 0.5, respectively.

The coupling and coordination degree can be divided into different types and levels according to the hierarchy. Referring to the mainstream interval division methods in academic circles [23], the coupling and coordination degree of the higher vocational education and the regional economic development is divided into 3 coordination hierarchies and 10 coordination levels, as shown in Table 2.

## 5. Empirical Results and Analysis

### 5.1. Comprehensive Evaluation of the Higher Vocational Education and the Regional Economic Development

**Figure 1:**
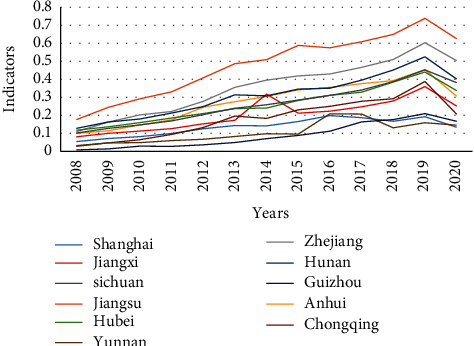
Comprehensive evaluation indicator of higher vocational education in the Yangtze river economic belt from 2008 to 2020.

**Figure 2:**
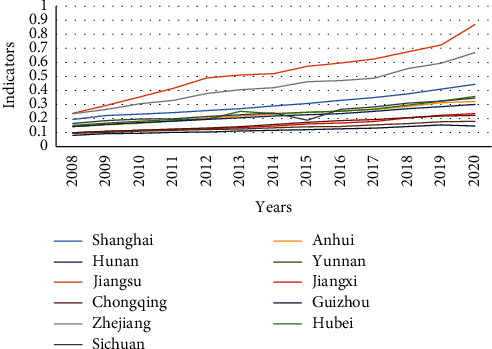
Comprehensive evaluation indicator of regional economic development in the Yangtze river economic belt from 2008 to 2020.

According to Equation (7), the comprehensive evaluation indicator of the higher vocational education system (*T*_1_) and the comprehensive evaluation indicator of the regional economic development system (*T*_2_) of the 11 provinces (cities) in the Yangtze River economic belt from 2008 to 2020 were calculated, and respectively, line graphs were drawn (see Figures 1 and 2).

According to Figure 1, higher vocational education in the Yangtze River economic belt is generally on the rise and has made great progress, which is correlated to the background of the times when China has been attaching great importance to the development of vocational education in recent years. Since 2005, China has successively enacted a series of policies, such as Decision of the State Council on Vigorously Developing the Vocational Education (The State Council [2005] No. 35), Decision of the State Council on Accelerating the Development of Modern Vocational Education (The State Council [2014] No. 19), and National Vocational Education Reform Implementation Plan (The State Council [2019] No. 4). These policies placed vocational education in a more prominent position in educational reform, innovation, economic, and social development, which vigorously boosted the connotative development of higher vocational education. However, 2019 witnessed the advent of an obvious turning point when the comprehensive evaluation indicator has reached its historical climax. Because of the impact of COVID-19 at the end of 2019 on the economic industry, the sharp decreases in internship training and employment scale of the students closely connected with enterprises have directly affected the number of recruited teachers, which is reflected in changes in teacher-student ratio and other indicators. Moreover, National Competition for Skills of Vocational Education in 2020, because of the pilot reform launched by Ministry of Education, dramatically decreased in competition items, competition teams, and number of winners compared with those in 2019, which has led to a decline in the indicator. In addition, the comprehensive evaluation indicator of the higher vocational education shows a relatively obvious phenomenon of provincial hierarchy. It is classified into three hierarchies by level: first hierarchy (Jiangsu, Zhejiang, and Hunan), second hierarchy (Anhui, Sichuan, Hubei, Jiangxi, Shanghai, and Chongqing), and third hierarchy (Yunnan and Guizhou).

According to Figure 2, from 2008 to 2020, the regional economic development system and the higher vocational education system in the Yangtze River economic belt have extremely similar development trend and a stable and upward growth trend. The average indicator of the regional economic development has risen from 0.1488 in 2008 to 0.3717 in 2020, a year-on-year increase of 149%. The average indicator of the comprehensive evaluation of the higher vocational education has risen from 0.0847 in 2008 to 0.3141 in 2020, a year-on-year increase of 270%. Apparently, the development speed of the higher vocational education is much higher than that of the regional economy. However, from the perspective of the development level, regional economy is generally higher than higher vocational education in the same period, and it also shows a relatively obvious phenomenon of provincial hierarchy, which is as follows: first hierarchy (Jiangsu, Zhejiang, and Hunan), second hierarchy (Anhui, Sichuan, Hubei, Jiangxi, Shanghai, and Chongqing), and third hierarchy (Yunnan and Guizhou).

To further measure the synchronicity of the higher vocational education system and the regional economic development system, a comparison was made between the annual average values of the comprehensive evaluation indicator of the higher vocational education system (*T*_1_) and the comprehensive evaluation indicator of the regional economic development system (*T*_2_) in the Yangtze River economic belt from 2008 to 2020, and the type of the coordinated development was calculated according to the calculation results (see Table 3). 6 provinces (cities), including Anhui, Jiangxi, Hubei, Hunan, Chongqing, and Sichuan, have the advanced higher vocational education, accounting for 54.5% of the total number of cities in the Yangtze River economic belt. 5 provinces (cities), including Shanghai, Jiangsu, Zhejiang, Yunnan, and Guizhou, have the lagged higher vocational education, accounting for 45.5% of the total number of cities in the Yangtze River economic belt. On the one hand, there exists major interprovincial differences in the development of the higher vocational education system and the regional economical development system. On the other hand, it also indicates that there exists a strong correlation between the two systems.

### 5.2. Analysis of Coupling and Coordination Degree of Higher Vocational Education and Regional Economic Development

According to equations (8) and (9), the coupling degree *C* and the coupling and coordination degree *F* of the higher vocational education system and the regional economic development system of the 11 provinces (cities) in the Yangtze River economic belt from 2008 to 2020 were calculated, respectively (see Table 4), and the coordination levels were divided (see Table 5).

According to the results of coupling degree, the coupling degree of the two major systems in Guizhou Province in 2008 and 2009 were 0.5691 and 0.6575, respectively, which were at a moderate coupling level, and the coupling degree of other provinces (cities) over the years was higher than 0.8, indicating a high coupling degree. From the perspective of the specific rankings, Jiang has the highest average coupling degree (0.9965), followed by Hubei (0.9961), and Guizhou has the lowest average coupling degree (0.8934). From the perspective of the annual average of the coupling coordination degree, most of the provinces have a relatively-low coupling and coordination degree. Out of the 11 provinces (cities), only Jiangsu (0.6979) and Zhejiang (0.6142) have the annual average values of the coupling and coordination degree higher than 0.6, which are in the primary coordination stage of the coordination type. Hunan (0.5029) is in the narrow coordination stage of the transition type. Sichuan (0.4932), Hubei (0.4923), Anhui (0.4929), Shanghai (0.4433), and Jiangxi (0.4140) are in the near imbalance stage of the transition type. Chongqing (0.3994) and Yunnan (0.3373) have the annual average values of the coupling and coordination degree lower than 0.4, and they are in the mild imbalance stage of the imbalance type. Guizhou (0.2991) in the last place is in the moderate imbalance stage of the imbalance type.

The empirical results show that the Yangtze River economic belt area of higher vocational education and regional economic development coupling general is on the rise since 2008 at a high level of coupling state. It shows that the two systems complement each other and depend on each other. The improvement of the development of higher vocational education becomes an important engine to promote the development of regional economy. The development of regional economy provides conditions and market demands for the continuous development of higher vocational education, and the high-quality coupling between higher vocational education and economic and social development needs long-term running-in to achieve, which is related to the adaptation of vocational education to the economy [24]. However, from the coupling coordination degree value, the overall situations of the coordinated development of the two are not ideal. From the perspective of the supply side of talent training, in the process of high-quality development of the industrial economy and high-end development of talent demand specifications, the higher vocational colleges are still relatively backward in specifications, types, methods, and other aspects of the talent training, which cannot match the requirements for high-end industries and industrial high-end development [25]. In addition, with the rapid development of emerging technologies, the setup and adjustment of some specialty groups obviously lag behind the speed of industrial development, and the strength of talent training is not enough to support the requirements of regional industrial transformation and upgrading, especially for strategic emerging industries [26]. It has led to various degrees of imbalance between the higher vocational education and the regional economic development in some provinces, and the coupling and coordination degree values of the two systems are low.

## 6. Conclusions and Recommendations

### 6.1. Conclusions

This research constructs a comprehensive evaluation indicator system for the higher vocational education system and the regional economic development system based on the panel data from 2008 to 2020, and it calculates and analyzes the coupling degrees and the coupling and coordination degrees of the higher vocational education and the regional economic development of the 11 provinces (cities) in the Yangtze River economic belt using the coupling and coordination degree model. The main conclusions are as follows:

1. From the perspective of comprehensive evaluation, the overall development level of the higher vocational education system and the regional economic development system in the Yangtze River economic belt has fluctuated and increased during the 13 years since 2008. The average value of the comprehensive evaluation indicators of the regional economic development system is slightly higher than that of the higher vocational education system. However, the higher vocational education system has been rapidly improved because of favorable policies, and its development growth rate is much higher than that of the regional economic development system.
2. From the perspective of coupling coordination degree, there is a good coupling interaction between the higher vocational education system and the regional economic development system in the Yangtze River economic belt. The two systems have been in a high-level coupling status over the years, however, the coordinated development of the two systems is not ideal. By the end of 2020, 73% of the provinces (cities) were in transition and imbalance, and only Jiangsu, Zhejiang, and Sichuan were in coordinated development. However, Chongqing, Guizhou, and Yunnan, which ranked the last three in the coupling coordination, belong to the upper reaches of the Yangtze River economic belt. The coupling coordination degree presents a gradient phenomenon of “high in the East and low in the west,” i.e., the more serious the imbalance is in the West.
3. From the perspective of the coordinated development type, only 6 provinces (cities) have the advanced higher vocational education, and the remaining 5 provinces (cities) have the lagged higher vocational education, indicating that the acceleration and drive of the higher vocational education in the Yangtze River economic belt for the regional economic development are not sufficiently prominent as a whole. The reasons for such incoordination are mismatch between the talent training scale of the higher vocational education and regional industrial development, inconsistency between specialty setup of higher vocation colleges and economic and industrial structures, and the fact that the talent training capabilities in some colleges lag behind the requirements of the regional economic development.

### 6.2. Recommendations

The Yangtze River economic belt is an important carrier for the coordinated development of the eastern, central, and western regions of China, and it has an important strategic position in the overall pattern of China's regional development. Promoting the coordinated development of the higher vocational education and the regional economy is an inevitable course and an important path to realize the high-quality development of the Yangtze River economic belt [27]. Therefore, in view of the current situation and problems of the higher vocational education and the regional economic development in the Yangtze River economic belt, the recommendations proposed are as follows:

1. Conform to the needs of industrial development and deepen the supply-side reform of the higher vocational talent training. The coupling and coordination problems of the higher vocational education and the regional economic development are essentially the problems of whether the development scale of higher vocational education and the quality of talent training match the needs of economy and industry, and whether it can effectively accelerate the high-quality development of economy and industry. In the stage of new normal development of economy and high-end development of industries, the higher vocational colleges shall link industries and enterprises to deeply participate in the cultivation of skilled talents and craftsmen [28]. On the one hand, the higher vocational colleges shall conduct regular research on the trends of economic development to closely meet the reform needs of key enterprises in the industry, take the establishment of a market-oriented technological innovation system as an important part of the colleges' innovation education system, organize enterprises' technical backbones and colleges' personnel in charge of specialty setup to extract the industry-college-research factors from the industrial demand side and the talent supply side, respectively, and jointly develop a talent training plan to promote the effective connection between the talent training chain and the industrial chain. On the other hand, the key enterprises in the industry shall share their new technologies, new industry standards, and new production norms with the higher vocational colleges from the perspective of cultivating reserve talents and undertaking social responsibilities, support the integration of the latest factors of the industry into the colleges' teaching standards and curriculum systems, jointly build training bases with the colleges, jointly build innovative research and development platforms, provide training resources and opportunities, and lead, participate in, and support the supply-side structural reform of high-quality technical and skilled talents in the higher vocational education.
2. Optimize the layout of higher vocational specialties and boost the adaptability of education to economy. The rationality of specialty setup and layout not only correlates to future and destiny of the higher vocational colleges but also directly affects the effectiveness of construction and services of regional economy [29]. From the perspective of the entire Yangtze River economic belt, governments at all levels shall strengthen linkage and coordination, keep a foothold in development orientation, industrial foundation, and resource endowments of the three major urban agglomerations in the upstream, midstream, and downstream regions of the Yangtze River economic belt, guide and manage the homogenization phenomenon of specialty setup in the higher vocational colleges from the institutional level, build an information-based quality supervision platform for the regionalized specialty construction of the higher vocational colleges, constrain or motivate the specialty setup of the higher vocational colleges in the whole region based on the platform, and conduct specialty construction quality management throughout the process. From the perspective of a single province in the Yangtze River economic belt, higher vocational education in various regions shall serve the regional economic development and optimize specialty groups and curriculum systems based on the college-running advantages and characteristics, thereby enhancing its ability to serve the economy. 6 provinces (cities) with advanced higher vocational education (Anhui, Jiangxi, Hubei, Hunan, Chongqing, and Sichuan) shall improve the construction mechanism of specialty groups that connect industries, serve the economy, and continuously upgrade the scale and quality of skilled talent training under the guidance of “high-level higher vocational college with Chinese characteristics and specialty construction plan.” 5 provinces (cities) with lagged higher vocational education (Shanghai, Zhejiang, Jiangsu, Yunnan, Guizhou, etc.) shall strengthen the investment in vocational education resources and financial resources, cultivate and import high-level “double-position teachers” and specialized teachers, and retain talents by improving the system and optimizing the environment to enhance the adaptability and contribution of vocational education to industrial development.
3. Strengthen regional overall coordination and accelerate the cluster development of regional higher vocational education. As far as any single province is concerned, its higher vocational education resources are limited. To reduce the provincial differences in the coordinated development of higher vocational education and the regional economy in the Yangtze River economic belt, it is necessary to break the administrative barriers between the provinces, promote reasonable sharing and flow of resources, such as training resources, industry experts, curriculum systems, and teaching staff, according to the correlation and similarity of the industrial structure in the Yangtze River Economic Belt and boost the overflow of superior vocational education resources in the central and eastern regions to the west regions [30]. In addition, it is necessary to accelerate the coordinated development plan of the urban agglomerations in the upstream and downstream regions of the Yangtze River Economic Belt, establish a new regional coordinated development pattern characterized by policy balance, industrial coordination, mobility of innovation factors, and close exchange of talents, and provide more preferential policies and resource support in vocational education for the provinces in the upstream regions of the Yangtze River economic belt, such as Yunnan and Guizhou, to narrow the differences in coupling and coordinated development between regions. At the specific implementation level, governments at all levels, colleges, industries, enterprises, society, etc., shall carry out multiparty linkages to boost the integration of production and education in “administration, teaching research, competition, training, and teaching,” ultimately form a benign interaction ecosystem characterized by the joint construction of economic belts, coordination of urban agglomerations, integration of industrial chain, and clustering of educational circle, and advance the coordinated development of higher vocational education and regional economy in the Yangtze River economic belt to a higher level and better quality.

## Figures and Tables

**Table 1:** Comprehensive evaluation indicator system and weights of higher vocational education system and regional economic development system.

System level	Criterion-level	Evaluation indicator level	Unit	Nature	Weight
Higher vocational education system	Education scale	Number of higher vocational colleges	College	Positive	0.0736
		Enrolments	Person	Positive	0.1117
		Number of students at colleges	Person	Positive	0.1290
	Education quality	National fiscal education funds	RMB 1000	Positive	0.1049
		Educational income	RMB 1000	Positive	0.0775
		Teacher-student ratio	—	Negative	0.0078
	Education achievement	Number of granted patents of higher vocational college	Patent	Positive	0.2136
		Number of awards in national competition for skills of vocational education (higher vocational college group)	Award	Positive	0.1919
		Number of national model (backbone) and “high-level higher vocational college with Chinese characteristics and specialty construction plan” higher vocational colleges	College	Positive	0.0900
Regional economic development system	Economic scale	Per capita gross regional product	RMB	Positive	0.1034
		Employees in all sectors of society	10,000 persons	Positive	0.0743
	Economic structure	Proportion of secondary industry in GDP	%	Positive	0.0293
		Proportion of tertiary industry in GDP	%	Positive	0.0716
	Economic benefit	Per capita income gap between urban and rural areas	RMB	Negative	0.0196
		GDP growth rate	%	Positive	0.0121
		Engel coefficient	%	Negative	0.0449
	Innovative development	Number of granted patents	Award	Positive	0.2581
		Internal expenditure of research & development (R&D) fund	RMB 10,000	Positive	0.1971
		Full-time equivalent of research & development (R&D) personnel	Man-year	Positive	1.1896

**Table 2:** Coupling and coordination degree level evaluation criterion.

Coordination hierarchy	Imbalance type (low hierarchy)				Transition type (medium hierarchy)		Coordination type (high hierarchy)			
Coordination level	Extreme imbalance	Severe imbalance	Moderate imbalance	Mild imbalance	Near imbalance	Narrow coordination	Primary coordination	Medium coordination	Good coordination	Superior coordination
*F* value	0.00–0.09	0.10–0.19	0.20–0.29	0.30–0.39	0.40–0.49	0.50–0.59	0.60–0.69	0.70–0.79	0.80–0.89	0.90–1.00

**Table 3:** Coordinated development types of “higher vocational education and regional economic development” in the Yangtze river economic belt.

Provinces	*T*_1_ annual average value	*T*_2_ annual average value	Difference between *T*_1_ and *T*_2_	Coordinated development type
Shanghai	0.1352	0.3015	−0.1663	Lagged higher vocational education
Jiangsu	0.4791	0.5289	−0.0498	Lagged higher vocational education
Zhejiang	0.3578	0.4288	−0.0710	Lagged higher vocational education
Anhui	0.2756	0.2292	0.0464	Advanced higher vocational education
Jiangxi	0.2025	0.1558	0.0468	Advanced higher vocational education
Hubei	0.2590	0.2395	0.0196	Advanced higher vocational education
Hunan	0.3092	0.2183	0.0909	Advanced higher vocational education
Chongqing	0.1836	0.1598	0.0238	Advanced higher vocational education
Sichuan	0.2616	0.2415	0.0201	Advanced higher vocational education
Yunnan	0.1061	0.1356	−0.0295	Lagged higher vocational education
Guizhou	0.0882	0.1171	−0.0289	Lagged higher vocational education

**Table 4:** Coupling degree and coupling and coordination degree of “the higher vocational education system and the regional economic development system” in the Yangtze river economic belt.

Years		Provinces										
		Shanghai	Jiangsu	Zhejiang	Anhui	Jiangxi	Hubei	Hunan	Chongqing	Sichuan	Yunnan	Guizhou
2008	C	0.8267	0.9889	0.9316	0.9843	0.9952	0.9917	0.9985	0.8282	0.9712	0.8668	0.5691
	F	0.3198	0.4518	0.4000	0.3451	0.2982	0.3653	0.3671	0.2295	0.3596	0.2333	0.1579
2009	C	0.8583	0.9957	0.9706	0.9882	0.9989	0.9945	0.9999	0.9189	0.9824	0.9294	0.6575
	F	0.3542	0.5173	0.4548	0.3643	0.3234	0.3881	0.4008	0.2669	0.3917	0.2628	0.1861
2010	C	0.8781	0.9952	0.9792	0.9981	0.9999	0.9980	0.9997	0.9572	0.9894	0.9214	0.8459
	F	0.3706	0.5665	0.4983	0.3944	0.3380	0.4120	0.4183	0.2946	0.4118	0.2694	0.2283
2011	C	0.9075	0.9935	0.9801	0.9997	0.9999	0.9998	0.9968	0.9899	0.9968	0.9464	0.8165
	F	0.3937	0.6083	0.5195	0.4233	0.3524	0.4343	0.4428	0.3288	0.4274	0.2884	0.2290
2012	C	0.9386	0.9959	0.9881	0.9956	0.9957	0.9992	0.9923	1.0000	0.9997	0.9570	0.8747
	F	0.4232	0.6682	0.5690	0.4726	0.3728	0.4485	0.4685	0.3633	0.4570	0.3006	0.2472
2013	C	0.9516	0.9997	0.9978	0.9931	0.9926	0.9996	0.9780	0.9876	0.9997	0.9787	0.9203
	F	0.4433	0.7058	0.6159	0.4948	0.3903	0.4942	0.5038	0.4085	0.4823	0.3185	0.2719
2014	C	0.9389	1.0000	0.9995	0.9907	0.9294	0.9999	0.9853	0.9972	0.9993	0.9877	0.9695
	F	0.4501	0.7171	0.6381	0.5153	0.4641	0.4873	0.5091	0.4119	0.4989	0.3383	0.3009
2015	C	0.9540	0.9999	0.9988	0.9858	0.9906	0.9975	0.9782	0.9898	0.9787	0.9816	0.9879
	F	0.4746	0.7616	0.6631	0.5364	0.4294	0.5131	0.5289	0.4470	0.4810	0.3411	0.3216
2016	C	0.9687	0.9998	0.9990	0.9860	0.9903	0.9946	0.9804	0.9888	0.9970	0.9825	0.9980
	F	0.5055	0.7649	0.6703	0.5476	0.4402	0.5291	0.5354	0.4637	0.5359	0.4154	0.3454
2017	C	0.9528	0.9999	0.9998	0.9846	0.9872	0.9948	0.9750	0.9833	0.9956	0.9897	0.9941
	F	0.5055	0.7848	0.6902	0.5611	0.4590	0.5444	0.5613	0.4807	0.5569	0.4232	0.3824
2018	C	0.9232	0.9998	0.9991	0.9878	0.9879	0.9914	0.9679	0.9849	0.9939	0.9936	0.9947
	F	0.5004	0.8134	0.7296	0.5791	0.4889	0.5807	0.5909	0.4951	0.5885	0.3825	0.3985
2019	C	0.9323	1.0000	1.0000	0.9830	0.9724	0.9884	0.9550	0.9599	0.9863	0.9985	0.9885
	F	0.5298	0.8550	0.7737	0.6137	0.5321	0.6145	0.6217	0.5390	0.6187	0.4096	0.4238
2020	C	0.8426	0.9867	0.9899	0.9997	0.9995	0.9996	0.9896	0.9993	0.9988	0.9944	0.9980
	F	0.4928	0.8584	0.7618	0.5601	0.4929	0.5890	0.5890	0.4627	0.6024	0.4021	0.3952
Annual average value	C	0.9133	0.9965	0.9872	0.9905	0.9876	0.9961	0.9844	0.9681	0.9914	0.9637	0.8934
	F	0.4433	0.6979	0.6142	0.4929	0.4140	0.4923	0.5029	0.3994	0.4932	0.3373	0.2991

**Table 5:** Coupling and coordination levels of the system of “higher vocational education and regional economic development” in the Yangtze river economic belt.

Years		Provinces										
		Shanghai	Jiangsu	Zhejiang	Anhui	Jiangxi	Hubei	Hunan	Chongqing	Sichuan	Yunnan	Guizhou
2008	C	High coupling	High coupling	High coupling	High coupling	High coupling	High coupling	High coupling	High coupling	High coupling	High coupling	Moderate coupling
	F	Mild imbalance	Near imbalance	Near imbalance	Mild imbalance	Moderate imbalance	Mild imbalance	Mild imbalance	Moderate imbalance	Mild imbalance	Moderate imbalance	Severe imbalance
2020	C	High coupling	High coupling	High coupling	High coupling	High coupling	High coupling	High coupling	High coupling	High coupling	High coupling	High coupling
	F	Near imbalance	Good coordination	Medium coordination	Narrow coordination	Near imbalance	Narrow coordination	Narrow coordination	Near imbalance	Primary coordination	Near imbalance	Mild imbalance
2008–2020 annual average value	C	High coupling	High coupling	High coupling	High coupling	High coupling	High coupling	High coupling	High coupling	High coupling	High coupling	High coupling
	F	Near imbalance	Primary coordination	Primary coordination	Near imbalance	Near imbalance	Near imbalance	Narrow coordination	Mild imbalance	Near imbalance	Mild imbalance	Moderate imbalance

## Data Availability

The experimental data used to support the findings of this study are available from the corresponding author upon request.
